# Gastric trichobezoar in an end-stage renal failure and mental health disorder presented with chronic epigastric pain: A case report

**DOI:** 10.1016/j.amsu.2020.08.021

**Published:** 2020-09-01

**Authors:** Aishath Azna Ali, Rajan Gurung, Zeena Mohamed Fuad, Muaz Moosa, Isha Ali, Ahmad Abdulla, Assikin Muhamad, Firdaus Hayati, Nicholas Tze Ping Pang

**Affiliations:** a Department of Surgery, Indira Gandhi Memorial Hospital, Male’, Maldives; b Department of Internal Medicine, Indira Gandhi Memorial Hospital, Male’, Maldives; c Department of Surgery, Faculty of Medicine and Health Sciences, Universiti Malaysia Sabah, Kota Kinabalu, Sabah, Malaysia; d Department of Community and Family Medicine, Faculty of Medicine and Health Sciences, Universiti Malaysia Sabah, Kota Kinabalu, Sabah, Malaysia

**Keywords:** Bezoars, Case reports, Psychiatric disorders, Trichobezoars

## Abstract

**Background:**

Gastric trichobezoar happens when there is an indigestible substance or food found in the gastrointestinal tract. It is a rare presentation which is usually associated with trichotillomania and trichopagia. The presentation may not be specific and is usually related to dyspepsia-like symptoms. In the worst-case scenario, this may cause gastric outlet or intestinal obstruction which eventually requires surgery.

**Case presentation:**

We present a 36-year-old lady with underlying end-stage renal failure (ESRF) and undiagnosed mental health issues who was treated for recurrent episodes of gastritis. Imaging modalities revealed intragastric foreign body ingestion which is consistent with gastric trichobezoar. She eventually underwent laparotomy and gastrotomy to remove the foreign body. Postoperatively, she was referred and followed-up by the psychiatric team.

**Conclusion:**

Gastric trichobezoar has strong associations with psychiatric disorders. With the co-existence of an ESRF, uraemia might contribute to the aetiology of the trichotillomania and trichophagia. Open surgery is the choice of definitive management especially if bezoars are larger. Should the recurrence be remitted, a biopsychosocial modality and regular haemodialysis is the most sustainable approach to ensure the behaviour does not persist.

## Highlights

• Depressive symptoms with concomitant end-stage renal failure might contribute to the occurrence of the trichotillomania and trichophagia, leading to gastric trichobezoar.
• Surgery is the mainstay of treatment in trichobezoar with complications.
• Referral to psychiatry is mandatory in addition to continuous medical treatment in patients with trichobezoar.

## 1 Introduction

Bezoar is a mass formed by indigestible substances or food found in the gastrointestinal tract [1]. It can be classified into its composition namely trichobezoar, phytobezoar, lactobezoar, foreign body bezoar and others [1]. Trichobezoar happens after ingestion of hair and food particles, commonly associated with mental health problems [1]. It is called gastric trichobezoar when it is found in the gastric region, however the moment it is extended distally to the small and large bowel, the pathology is called Rapunzel syndrome [1]. This may cause complications such as a gastric outlet or intestinal obstruction as well as perforated viscus. Symptoms may be missed in earlier stages as they are non-specific. However, a further social history is essential in order to elicit the associated mental health issues that might cause repetition of the behaviour. Despite successful diagnosis and surgery, treatment of underlying illness is important. A referral to a psychiatrist is paramount so that appropriate interventions can be carried out to avoid recurrence. Herein, we report a 36-year-old lady with end-stage renal failure (ESRF) and undiagnosed mental health issues, diagnosed as gastric trichobezoar after complaining of recurrent episodes of gastritis. This work has been reported in line with the SCARE criteria [2].

## 2 Case report

A 36-year-old Maldivian female was evaluated under the Department of Internal Medicine for heartburn, bloating and abdominal pain. She claimed to have occasional epigastric pain which was presumed to be gastritis and hence was managed with medication. The bloating sensation had started a few months prior and occasionally was associated with vague crampy abdominal pain. There was no specific aggravating or relieving factors. She had early satiety for the past few weeks. There were no altered bowel habits and constitutional symptoms. She had no family history of malignancy.

She has an underlying ESRF on regular dialysis. There was no history of previous surgery except for arteriovenous fistula. However, on further probing, she revealed that she always felt stressed, and had a history of depressive symptoms which had not been properly diagnosed or treated. She further admitted to having a tendency of pulling out (trichotillomania) and eating her own hair (trichophagia). General examination was unremarkable. She was well built with no signs of dehydration or anaemia. Abdominal examination revealed a hard, well defined and mobile epigastric mass. There was tenderness but no guarding or rigidity elicited. The succussion splash was negative. The bowel sounds were audible.

**Fig. 1:**
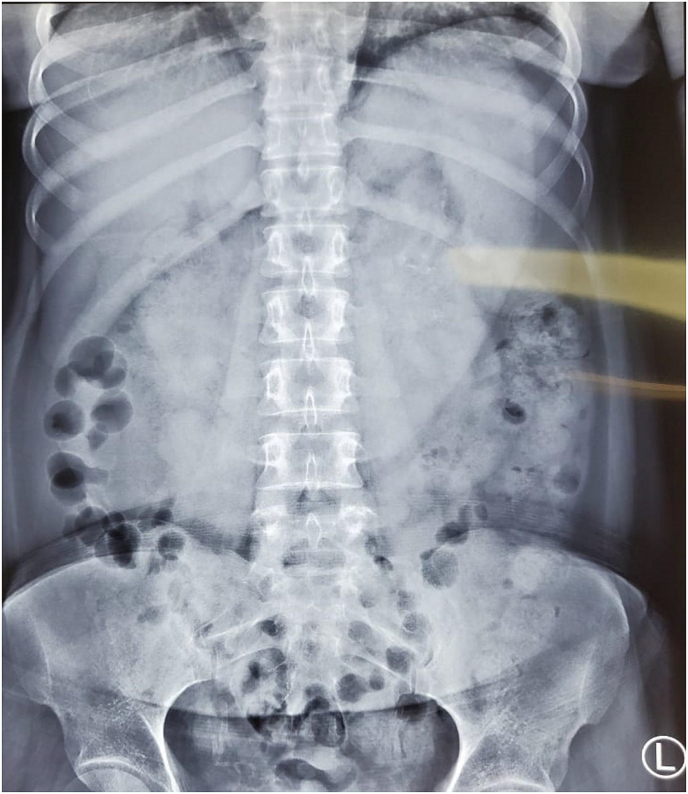
A plain abdominal radiograph showing a sizable soft tissue density with prominent stomach outline at left hypochondial region.

**Fig. 2:**
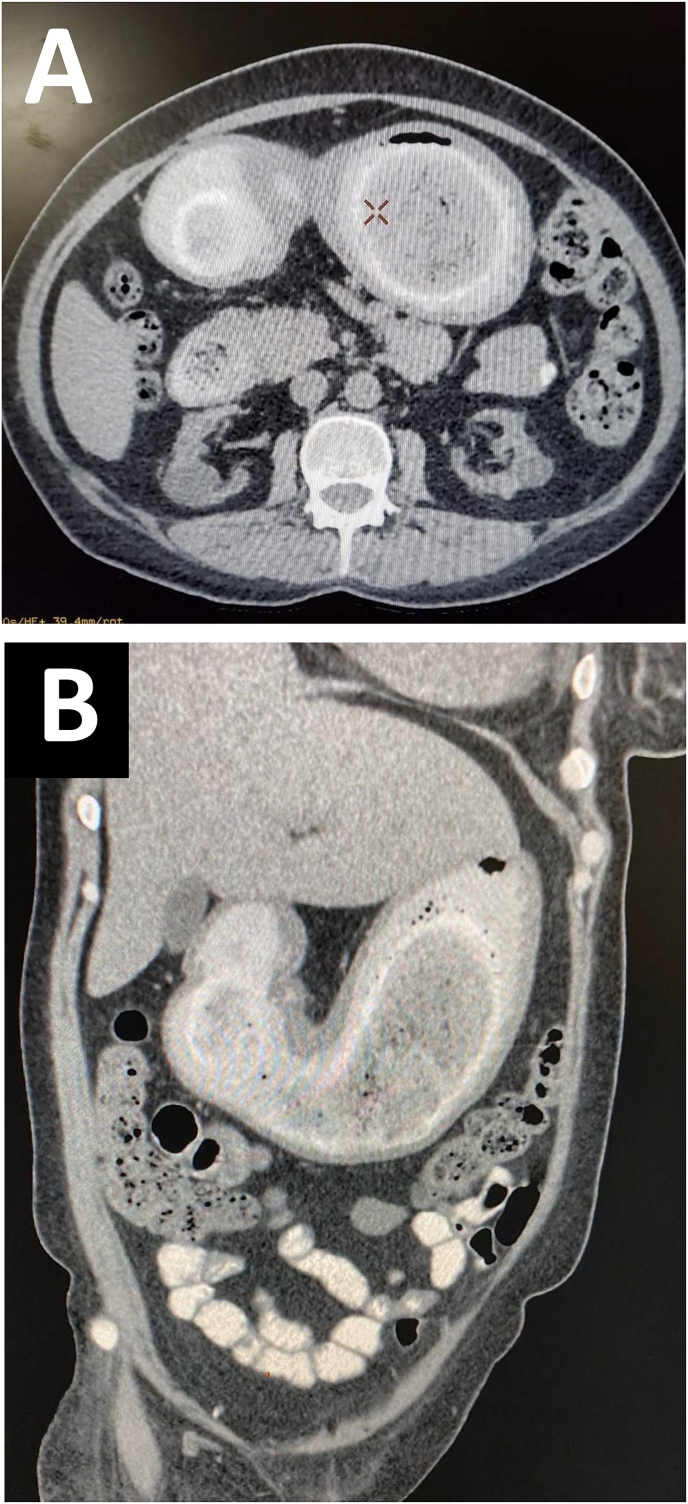
CT at axial (A) and coronal view (B) showed a bezoar in the stomach extending to the second part of the duodenum as outlined by contrast.

Laboratory investigations were within normal limits except for raised urea and creatinine owing to the ESRF. Her renal profile was elevated with serum urea was 72.76 (normal: 15.0–40 mg/dL) and creatinine was 6.38 (normal: 0.57–1.11 mg/dL). Plain abdominal radiograph (Fig. 1) showed a sizable soft tissue density with prominent stomach outline at the left hypochondrium. Contrasted computed tomography (CT) scan (Fig. 2A and B) was done which revealed a dilated, distended stomach with the intraluminal mass taking the shape of the stomach and extending up to the 2nd part of duodenum. The mass had a hyperdense outline with heterogeneous texture which mixed with air. The radiologic features were suggestive of intragastric foreign body ingestion.

**Fig. 3:**
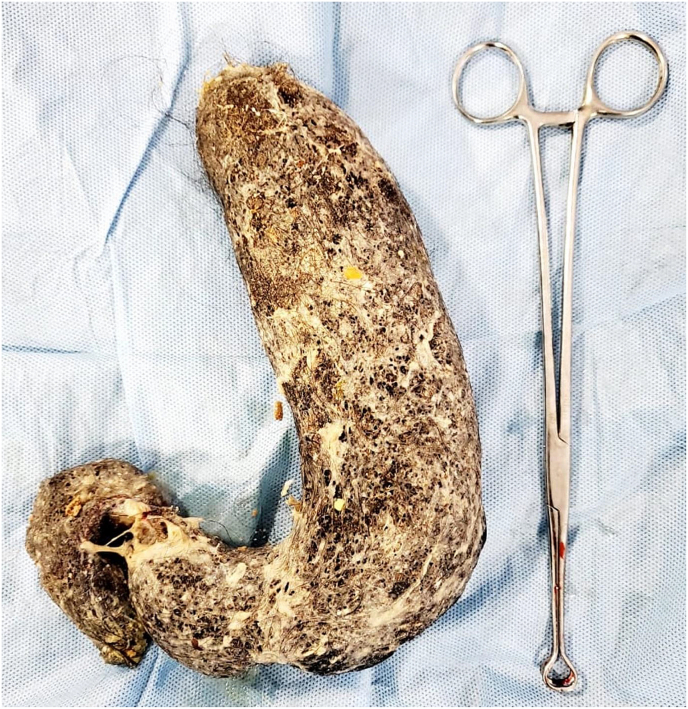
A stomach-shaped trichobezoar with a tapering tail extending into the small bowel is surgically removed *en bloc*.

She was preceded for exploratory laparotomy with gastrotomy and removal of the trichobezoar. The mass was removed en bloc. It weighed 720 gm (Fig. 3). Postoperatively, patient recovery was uneventful and she was discharged well after consultation with psychiatry team and series of haemodialysis.

## 3 Discussion

“Bezoar” is etymologically derived from the Arabic “bedzher” or “bezehr” which dates from 2000 years ago, meaning antidote or counter-poison. Historically, it was described as a greenish hard mass found inside the stomach of Syrian goats, which was believed to be a treatment of poison [3]. Trichobezoar had been recognized as early as autopsy reports in 1779 [3]. It may happen to both younger age groups and adults. However, both of these groups are usually associated with psychiatry illness and sometimes the former one related to congenital health problems. In younger age groups, psychiatry-related mood or anxiety disorder can be contributed by background social history such as parenting or sometimes environment in school [4,5]. Sometimes congenital-related conditions especially born prematurely with sensorineural hearing loss or cerebral palsy may contribute indirectly to this illness [6,8]. In adults, this may be associated with trichotillomania. Trichotillomania is a condition whereby patients are anxiously pulling their hair to get relief or satisfaction [7]. This is followed later by the compulsive act of trichophagia. This may result due to a depression secondary to the underlying poor social background or underlying disease. In our case, the patient suffers from ESRF with haemodialysis in which uraemia itself can complicate the psychological disorder. Our patient showed evidence of high levels of serum urea which could be the contributing factor of the mental health issues.

Sometimes it is difficult to diagnose trichobezoar because of the non-specific symptoms. Younger age groups such as paediatric patients are usually thought to have acute gastroenteritis or parasitic infection which is treated conservatively [11]. While in the adulthood, symptoms are usually vague as they may manifest as dyspepsia, pancreatitis or sometimes be thought to have malignancy [9]. The clinical presentations of definitive diagnosis usually present in various forms and severity. In our case, she presented as chronic heartburn, bloating, and gastritis-associated symptoms, which were not improved. However, in a worse scenario, it may manifest as more severe and life-threatening circumstances. They may present as upper gastrointestinal bleeding, biliary obstruction, intestinal obstruction or peritonitis [9,[12], [13], [14]]. In addition to associated psychiatry and the above clinical manifestations, some “clues” associated with this syndrome include halitosis which is due to bacterial colonization [10]. Diffuse thinning jagged hair or alopecia may also be a clue to this condition [7].

Imaging modalities help in establishing the diagnosis. Simple imaging such as abdominal radiograph can reveal a soft tissue density outlining the stomach or a J-shaped distended stomach with air-fluid level seen in the second part of duodenum. Ultrasound shows an echogenic curvilinear mobile shadow in the stomach with dense posterior acoustic shadow [15]. While CT abdomen may show imaging character of well-circumscribed, non-homogenous composed of concentric whorls of different densities with air enmeshed on it [5].

Approach for definitive treatment depends on location, consistency and size of trichobezoar. This may include extracorporeal wave lithotripsy, laser ignited mini-explosive technique, intragastric enzymes administration (pancreatic lipase, cellulose), metoclopramide, acetylcysteine, and endoscopic removal [5]. Endoscopic management may have a role in the management of this patient. However, almost all dense and large trichobezoars might be failed by endoscopic removal alone. Open surgery is a better choice as it may have a shorter duration and provides a more assuring and complete evacuation of the trichobezoar. Open gastrostomy and retrieval with synchronized milking of the distal length of the bezoar are the techniques of retrieval [5]. Laparoscopy, on the other hand, may have a role in reducing complications such as surgical site infection, atelectasis, and extensive scar. It can be carried out with the presence of relevant expertise. During laparoscopy, the bezoars are fragmented into pieces by polypectomy snare and argon plasma coagulation and then retrieved [10].

Once the patient is stable, if indicated, referral to a psychiatrist is important as the bezoar will otherwise recur. The trichotillomania can be the pathology proper or can be the tip of the iceberg of other psychiatric pathology including depressive or anxiety disorder, eating disorders, or more rarely, frank psychosis. Hence, trichotillomania should not be seen as an end, but rather, a means, and underlying psychosocial issues must be teased out gently. This is because correction of the psychiatric illness itself will not suffice if the social issues that predispose or precipitate the hair-eating behaviour are not attended to holistically. For the treatment of the underlying psychiatric issue, given the complexity of trichotillomania presentations, a biopsychosocial integrated model is preferred, with behavioural therapy, other forms of psychotherapy, and antidepressant or antipsychotic medications best combined to attain recovery [7]. The efficacy of both cognitive behaviour therapy and selective serotonin reuptake inhibitors in treating underlying depressive and anxiety disorders are reasonably high, and hence efforts must be taken to banish or reduce all self-stigma or enacted stigma towards the possibility that trichobezoar in patients has an underlying psychiatric aetiology.

In conclusion, gastric trichobezoar is a rare form of medical illness that can lead to gastric outlet obstruction and chronic gastritis especially among ESRF patients. Due to its rarity, thorough history taking focusing on social, medical and sometimes psychiatric symptoms is very important to elicit the association of this illness. Abdominal radiograph and CT scan are helpful in establishing a definitive diagnosis which mandates surgical removal of the trichobezoars. Follow-up not only focuses on post-operative care solely but also concerns on psychiatry referral and routine haemodialysis, which may indirectly prevent a recurrence.

## Ethical Approval

Not related as this is a case report

## Source of funding

Not available

## Authors contributions

AAA - manuscript preparation

RG - involvement in managing the patient

ZMF - data collection.

MM - involvement in managing the patient

IA - literature search

AA - involvement in managing the patient

AM - manuscript preparation

FH - final review

TPNP - preparation of psychiatric input

## Registration of research studies

This is a case report. No human participants were involved.

## Guarantor

FH

## Consent

Consent was obtained from the patient

## Provenance and peer review

Not commissioned, externally peer-reviewed.

## Declaration of competing interest

The authors declare that no relevant or material financial interests exist.

## Appendix

ESRF: end-stage renal failure.
CT: computed tomography.
